# Rare bacterial subcommunity drives nutrient cycling in phyllosphere habitat of evergreen conifers

**DOI:** 10.1128/spectrum.00518-25

**Published:** 2025-07-15

**Authors:** Bing Li, Mingyang Fu, Guangze Jin, Zhili Liu

**Affiliations:** 1 School of Ecology, Northeast Forestry University 47820, Harbin, China; 2 Key Laboratory of Sustainable Forest Ecosystem Management-Ministry of Education, Northeast Forestry University 47820, Harbin, China; 3 Northeast Asia Biodiversity Research Center, Northeast Forestry University 47820, Harbin, China; University of the Philippines Los Baños, Laguna, Philippines

**Keywords:** phyllosphere bacteria, rare taxa, needle age, nutrient cycling, evergreen conifers, functional traits

## Abstract

Phyllosphere bacteria are crucial for pathogen resistance, stress tolerance, and productivity maintenance of host plants and further have potential effects on ecosystem functions. However, whether and how assembly patterns of both abundant and rare subcommunities changed across needle age cohorts, and their relative contributions to nutrient cycling in phyllosphere habitat of evergreen conifers are still unclear. Here, we examined both phyllosphere abundant and rare bacterial subcommunities in three needle age cohorts of the representative evergreen conifers in mixed broadleaved-Korean pine forests throughout Northeast China. We found that dispersal limitation and ecological drift dominated abundant and rare subcommunities, respectively. Deterministic assembly gradually increased with needle aging, which was mainly attributed to the increased needle chemical defense traits such as flavonoids. We further showed that multiple nutrient cycling in phyllosphere habitat was regulated by the rare subcommunity, closely related to the variations in diversity and composition. Collectively, our findings facilitate us in clarifying the mechanisms underlying the formation and succession of phyllosphere bacterial subcommunities across needle age cohorts and emphasize the vital role of rare subcommunities in promoting ecosystem functions.

**IMPORTANCE:**

Host-associated microbial communities are critical for host health. However, the relative importance of abundant and rare subcommunities in driving nutrient cycling in phyllosphere habitat across needle age cohorts of evergreen conifers remains unknown. Here, we showed the different assembly mechanisms of abundant and rare subcommunities and emphasized the ecological role of rare subcommunities in promoting ecosystem functions. This is useful for understanding the formation and succession dynamics of plant microbiome to advance future applications with microbial agents to sustainable productivity and reduce diseases.

## INTRODUCTION

The aerial surface of leaves, expressed as the “phyllosphere,” is an important and universal microbial habitat in terrestrial ecosystems (1). Phyllosphere microorganisms have been proven to play an important role in maintaining host health and mediating the relationships between plant diversity and ecosystem function (2). Previous studies have indicated that the assembly of phyllosphere microbial communities is jointly governed by the environmental factor and host identity (3–5). For instance, the continuous input of microbial taxa originating from surrounding environments, such as rain, atmospheric, soil, and neighboring plants, is prevalent, especially for open phyllosphere environment (6). Therefore, initial microbial communities with relatively low diversity in newly formed leaves can be randomly populated by recently arrived taxa, where stochasticity dominated the community assembly (6, 7).

Moreover, the successful colonization of microbial taxa in phyllosphere habitat is significantly influenced by leaf functional traits (8, 9). Leaf economic traits (e.g., N and P content) can determine the exudation potential of carbohydrates and nutrients from the inside of the leaf to the surface (10, 11). In addition, leaf chemical defense traits (e.g., phenolic compounds and tannins) play an important role in mediating plant-microbe interactions through serving as signaling molecules to recruit specific microorganisms or as antibacterial substances to filter microorganisms (12). Therefore, leaf functional traits are regarded as important predictors for the assembly processes of phyllosphere microbial community (13). However, microbial communities typically present a skewed abundance distribution manifested as a minority of dominant species and a majority of low-abundance species that are named as “rare biosphere” (14, 15). Previous studies have shown that there were substantial differences in the assembly processes between abundant and rare subcommunities. For instance, Xiong et al. (16) indicated that the rare fungal subcommunity in crop phyllosphere was more sensitive to host selection and mainly regulated by deterministic assembly, while the abundant subcommunity was mainly governed by stochastic assembly. Nevertheless, whether and how needle economic traits and chemical defense traits regulate assembly processes of abundant and rare subcommunities in phyllosphere habitat of evergreen conifers still remain unexplored.

Recently, needle age has been proven to induce variations in needle functional traits of evergreen conifers (17). For instance, mature needles generally have higher photosynthetic capacity and leaf dry matter content than newly formed needles, while the specific leaf area and leaf nitrogen content (N_mass_) significantly decrease with needle aging, notably because of the accumulation of non-nitrogen compounds such as cell walls (18, 19). Additionally, intense lignification of cell wall structure in older needles can reduce porosity, which may, in turn, limit carbon and nutrients exudation to needle surface (18). These changes in functional traits of needles with different ages will lead to varying environmental pressures influencing the composition of phyllosphere microbial communities (20). On the other side, the longer accumulation period of bacterial taxa with needle aging may lead to increased microbial diversity (6). Moreover, the thicker epidermis composed of long-chain hydrocarbons in older leaves may create distinct ecological niches and provide multi-protection for phyllosphere microbes against dehydration, UV, and other abiotic stresses such as heavy wind and rain compared to young leaves (21). As a result, the microbial community thriving on young needles may differ significantly from that found on mature needles, leading to age-dependent shifts in microbial diversity and function. However, there are still knowledge gaps regarding the assembly dynamics of phyllosphere abundant and rare subcommunities across needle age cohorts, as well as their relationships with needle functional traits.

Understanding the microbial ecological roles in driving nutrient cycling in phyllosphere habitat has become a hotspot in ecological research in recent years (22). Some microbial taxa surviving on leaf surface have been proven to take part in the metabolism of carbohydrates and amino acids (13). This is helpful for enhancing the adaptability of the entire community to low nutrient availability and climate fluctuations and further contributes to host plant health through mediating nutrient absorption and increasing pathogen resistance (23, 24). Evidence is mounting that the abundant and rare taxa have distinct ecological roles (15, 25). Abundant taxa are believed to be more inclined toward maintaining a larger number of functions (26). Contrarily, rare taxa are often associated with efficient utilization on unusual resources such as nitrogen (N_2_), sulfate, and methane (27, 28). Nevertheless, increasing research highlights the crucial role of rare subcommunities in driving ecosystem multifunctionality and biodiversity-ecosystem functioning relationships (16, 29). Accordingly, exploring the relative contributions of abundant and rare subcommunities to nutrient cycling in phyllosphere habitat will enhance our understanding of the importance of phyllosphere biodiversity in maintaining forest ecosystems.

In this study, we aim to (i) explore the assembly patterns of both abundant and rare subcommunities across needle age cohorts and their associations with needle functional traits and (ii) determine the relative contribution of abundant and rare subcommunities to nutrient cycling in phyllosphere habitat. We addressed these knowledge gaps using the amplicon sequencing data of phyllosphere epiphytic bacteria across 252 needle samples corresponding to three age cohorts (i.e., “young,” “mature,” and “old”) from three representative evergreen conifers (*Pinus koraiensis*, *Picea koraiensis,* and *Abies nephrolepis*) in five natural mixed broadleaved-Korean pine forests throughout Northeast China. Moreover, we determined the microbial functional genes with metagenomic sequencing. We first hypothesized (*H*_1_) that both abundant and rare subcommunities were greatly governed by deterministic assembly, notably rare subcommunity in old needles, while stochastic assembly was more important in young needles, especially for abundant subcommunity. Specifically, we expected that the increased deterministic assembly with needle aging was closely related to the needle economic traits rather than chemical defense traits. Given the universally positive effects of biodiversity on ecosystem multifunctionality across contrasting ecosystems (30). We further hypothesized (*H*_2_) that rare subcommunities contributing more biodiversity performed better in regulating nutrient cycling in phyllosphere habitat compared with abundant subcommunities.

## MATERIALS AND METHODS

### Study site description

This study was carried out in five mixed broadleaved-Korean pine forests located in Shengshan (SS), Fenglin (FL), Liangshui (LS), Muling (ML), and Changbaishan (CBS) natural reserves throughout Northeast China (126.78°−128.12°E, 42.32°−49.48°N) (Table S1; Fig. S1), representing one of the major temperate mixed forest regions in the world (31). The deciduous broadleaved species are dominated by *Betula platyphylla* Suk., *Acer pictum subsp. mono (Maxim.) H. Ohashi,* and *Quercus mongolica* Fisch. ex Ledeb, while *Pinus koraiensis* Siebold & Zucc., *Picea koraiensis* Nakai, and *A. nephrolepis* (Trautv. ex Maxim.) Maxim dominated the evergreen conifers (32, 33).

### Sample collection and chemical analyses

For each site, we randomly selected six adult individuals per species with diameter at breast height (DBH) of 35–70 cm for *Pinus*, 20–45 cm for *Abies,* and 30–60 cm for *Picea*. The distance between any two individuals was at least 10 m to reduce the interference of spatial autocorrelation (32). We divided needles into three age cohorts according to the polycyclic shoots (34), including young and mature needles those growing on 1-year-old and 3-year-old branches, respectively. Moreover, the needles growing on 5-year-old and older branches were taken as old needles, as 5-year-old needles are scarce in quantity. We collected about 50 g of fresh and healthy needles per age cohort of each individual with surface-sterilized shears from four branches growing in subcanopy (about 8–12 m above the ground) and extending toward east, south, west, and north (1). There are a total of 252 needle samples in this study (5 sampling sites × 3 evergreen conifers ×  6 individuals ×  3 needle age cohorts) (*Abies* were not found in the SS sampling site). All needle samples were transported to the laboratory directly after collection, and they were randomly divided into two parts: one subsample was used for phyllosphere microbial DNA extraction, and the other subsample was used for needle chemical analysis.

Needle functional traits, including economic traits such as total carbon (TC), total nitrogen (TN), and total phosphorus (TP), as well as chemical defense traits such as total phenols, tannins, and flavonoids, were measured. Briefly, needles were first oven-dried at 60°C for 48 hours. Homogenized needles were then ground with a ball mill for further analyses. TC and TN were assessed using an automatic elemental analyzer (Vario MACRO cube, Germany). TP was extracted with H_2_SO_4_-HClO_4_ and then evaluated with the molybdenum blue method. Total phenols and tannins were evaluated using Folin-Ciocalteau reagent following Makkar (35) after extracting phenols with 75% acetone, and tannic acid was taken as a standard. Flavonoids were measured with NaNO_2_-AI(NO_3_)_3_-NaOH complexometry according to Kim et al. (36), and rutin was used as a standard.

### Phyllosphere microbial DNA extraction and sequencing

Phyllosphere epiphytic microorganisms were collected from 24 g needles using sterilized ultra-pure water (needle:water = 1:10) according to Bodenhausen et al. (37). Briefly, ultrasonic vibration extraction was performed for 1 minute, vortex for 10 seconds, and then these procedures were repeated once. The suspensions were merged and filtered with a 0.22 µm filter, and the filter was then used for microbial DNA extraction. Microbial DNA extraction was conducted with the FastDNA Spin Kit for Soil (MP Biomedicals, USA), following the standardized method described in the manufacturer’s protocol. Primers 515F and 907R were used to amplify the V4–V5 region of bacterial 16S rDNA. Negative controls without template were included to assess the potential contamination during PCR amplification. PCR products were sequenced with the Illumina MiSeq PE300 platform. Sequences were quality filtered (fastp v0.19.6) and merged (FLASH v1.2.11). Specifically, low-quality reads with length < 50 bp or with a quality value < 20 or having N bases were filtered. Sequences with overlap longer than 10 bp were merged, and mismatch density in the overlap region was set to 0.2. The full-length sequences were then de-noised with DADA2 in Qiime2 v2020.2 to obtain amplicon sequence variants (ASVs) (38). A total of 8,818,936 high-quality paired-end reads were kept. After filtering out the sequences aligned to chloroplast and mitochondrial, 8,215,024 reads were left. We then rarefied the sequences of each sample to a minimum number of sequences (6,730) in order to minimize the effects of sequencing depth. Taxonomic assignment was conducted with the Naive Bayes classifier in Qiime2 against the Silva 138 database at a 0.7 confidence threshold. We obtained a total of 60,770 ASVs across all 252 needle samples. The phylogenetic tree was constructed with IQ-TREE v1.6.8 using the maximum likelihood method (39).

### Metagenomic sequencing

To further clarify the potential functional profiles of phyllosphere microbial communities, metagenomic sequencing was performed with the Illumina NovaSeq platform for 76 DNA samples from young and mature needles that were collected from SS, LS, and CBS sampling sites (20 DNA samples were removed due to their insufficient DNA content to meet the requirements for library construction). These two needle age cohorts and three sampling sites have been proven to induce a wide range of changes in needle functional traits and therefore are expected to arise greater effects on the composition and function of phyllosphere microbial communities (19). Raw sequences were quality trimmed with fastp v0.20.0, and 6.0 Gb of clean data were retrieved for each DNA sample. A non-redundant gene catalog was generated using CD-HIT v4.6.1 with sequence identity and coverage at a 0.90 level (40). High-quality reads were aligned to non-redundant gene catalogs using SOAPaligner v2.21 with a 0.95 identity. The functional profiles (i.e., KEGG Orthology) were obtained by aligning representative sequences to the KEGG database using Diamond v0.8.35 with an *e*-value cutoff of 10^−5^. We used transcripts per kilobase of exon model per million mapped reads (TPM) to normalize gene abundance (41).

### Definitions of the abundant and rare taxa

The rare taxa are regarded as a combination of rare viable and dormant taxa observed in prescriptive environmental samples at specific timepoints, and as such, the definition of the rare taxa has been arbitrary (42). Currently, numerous published literatures on different ecosystems (e.g., marine, agricultural field, dryland, steppe, and wetland) prefer to utilize the thresholds of relative abundance to distinguish between abundant and rare taxa, such as defining the abundant taxa as mean relative abundances > 0.1% and the rare taxa as mean relative abundances < 0.01% (43–47). Hence, the same method was used in this study to designate the abundant and rare taxa as the ASVs with mean relative abundance >0.1% and <0.01% of total sequences across 252 samples, respectively, whereas those ASVs with mean relative abundances between 0.01% and 0.1% were defined as intermediate taxa. To avoid random effects, we further discarded the ASVs that occurred in less than 10 samples.

### Statistical analysis

We calculated Shannon diversity and Faith’s phylogenetic diversity (PD) to estimate the α-diversity of both abundant and rare subcommunities with the “vegan” and “picante” R packages (48, 49). To assess whether α-diversity changed across needle age cohorts and different subcommunities, the Kruskal-Wallis test was used with the “scheirerRayHare” function in the “rcompanion” R package as the data did not meet the analysis of variance (ANOVA) (50). β-diversity was estimated based on the Bray-Curtis distance and Weight-UniFrac distance, respectively, and then visualized using principal coordinate analysis (PCoA). Permutational multivariate analysis of variance (PERMANOVA) was used to estimate whether abundant and rare subcommunities were significantly influenced by needle age with the “vegan” and “phyloseq” R packages (48, 51). Redundancy analysis (RDA) was performed to determine the relative contributions of needle functional traits to abundant and rare subcommunities. We conducted a *t*-test to determine the difference in abundance of functional genes between young and mature needles, and when data did not meet the ANOVA, the Kruskal-Wallis test was used. Differential analysis was performed using the “edgeR” package with the generalized linear models approach to identify the biomarker taxa that are significantly enriched in young or mature needles at the threshold of log_2_(fold change) >0 and FDR-adjusted *P* < 0.05 (52).

The “null model” was used to estimate the assembly processes of both abundant and rare subcommunities according to Stegen et al. (53) with the “icamp” R package (54). Briefly, β-nearest taxon index (βNTI) > 2 indicates that heterogeneous selection (HeS) dominated the community, while βNTI < −2 represents homogeneous selection (HoS); |βNTI| < 2 with Bray-Curtis-based Raup-Crick (RC_bray_) > 0.95 or RC_bray_ <−0.95 indicates the dominant effects of dispersal limitation (DL) or homogeneous dispersal, respectively, and |RC_bray_| < 0.95 represents ecological drift (ED). The Mantel test was conducted to explore the correlation between community assembly and needle functional traits based on the βNTI values and Euclidean distance matrices of each trait.

The structural equation model (SEM) was constructed in order to distinguish the microbial drivers of nutrient cycling in phyllosphere habitat using the Amos 17.0 software (SPSS Inc., Chicago, IL, USA). We assumed that needle aging would affect the diversity and composition of abundant and rare subcommunities (55), which could further lead to significant variations in functional gene abundance due to their differences in metabolic capacities (15, 25). The initial model with all the priori hypothesized relationships was given in Fig. S2. Needle age gradient was regarded as an exogenous variable; diversity and composition of abundant and rare subcommunities were treated as endogenous variables; nutrient cycling index, calculated as average abundance of functional genes involved in C, N, P, and S cycling, was considered as a response variable. Data were Box-Cox transformed using the “car” R package to improve the normality in order to make the model more robust (56). The model quality was assessed using the *χ* value (*χ*^2^), degrees of freedom (df), *P*-value, root mean square error of approximation (RMSEA), and goodness-of-fit (GFI) (57).

## RESULTS

### Needle functional traits and bacterial subcommunities changed across needle age cohorts

**Fig 1:**
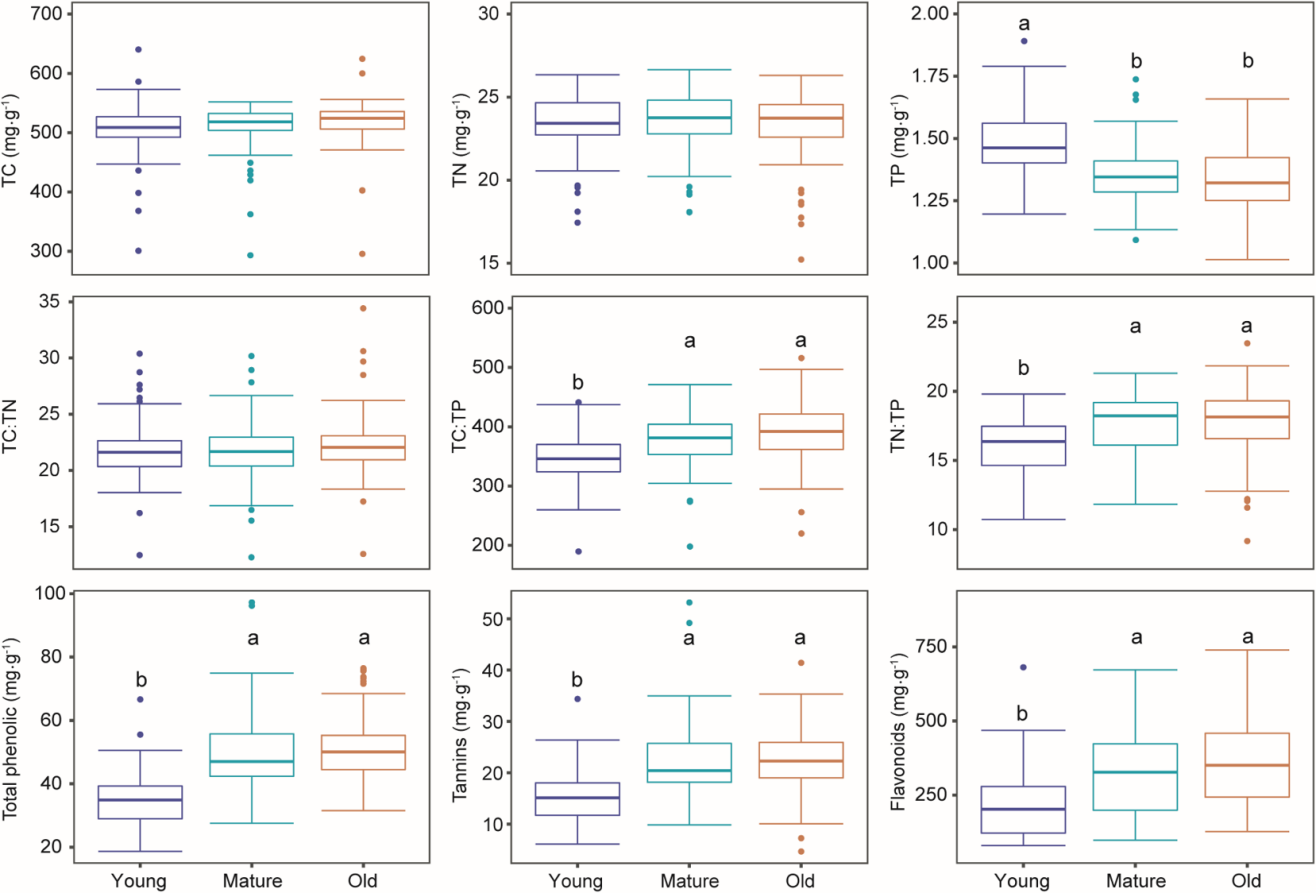
Needle functional traits across needle age cohorts. Different lowercase letters indicated significant differences at *P* <  0.05. TC, total carbon; TN, total nitrogen; TP, total phosphorus.

**Fig 2:**
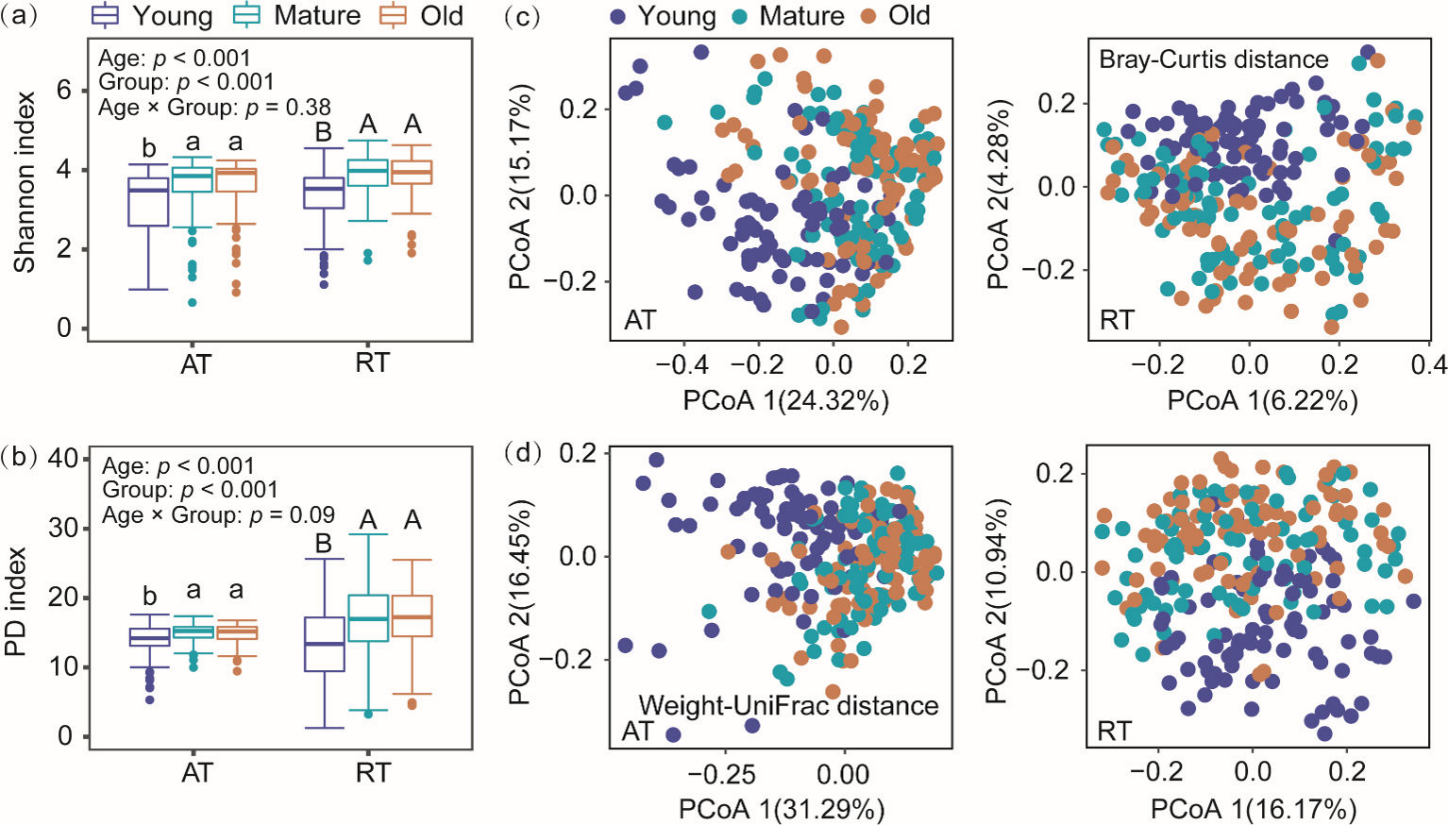
Shannon diversity (**a**), phylogenetic diversity (**b**), and principal co-ordinates analysis (PCoA) of abundant (AT) and rare (RT) subcommunities composition based on the Bray-Curtis distance (**c**) and Weighted-UniFrac distance (**d**). Different uppercase and lowercase letters indicated significant differences at *P* <  0.05.

We found that TC and TN did not change, while TP decreased with needle aging (Fig. 1). It resulted in increased values of TC:TP and TN:TP over time (Fig. 1). Total phenols, tannins, and flavonoids showed similar trends with higher values in mature and old needles compared with young needles (Fig. 1). When investigating the phyllosphere bacterial subcommunities, we found that the Shannon diversity and PD of both abundant and rare subcommunities were the lowest in young needles, followed by mature and old needles (Fig. 2a and b). In addition, the rare subcommunities were more diverse than the abundant subcommunities (*P* < 0.001) (Fig. 2a and b). The community composition of both abundant and rare subcommunities was significantly affected by needle age (Table S2; Fig. 2c and d). Particularly, we found that abundant subcommunities originating from different needle age cohorts were largely separate along the PCoA1 axis, and rare subcommunities were largely separate along the PCoA2 axis. Therefore, we used PCoA1 and PCoA2 scores to represent variations in community composition of abundant and rare subcommunities, respectively (Fig. 2c and d).

**Fig 3:**
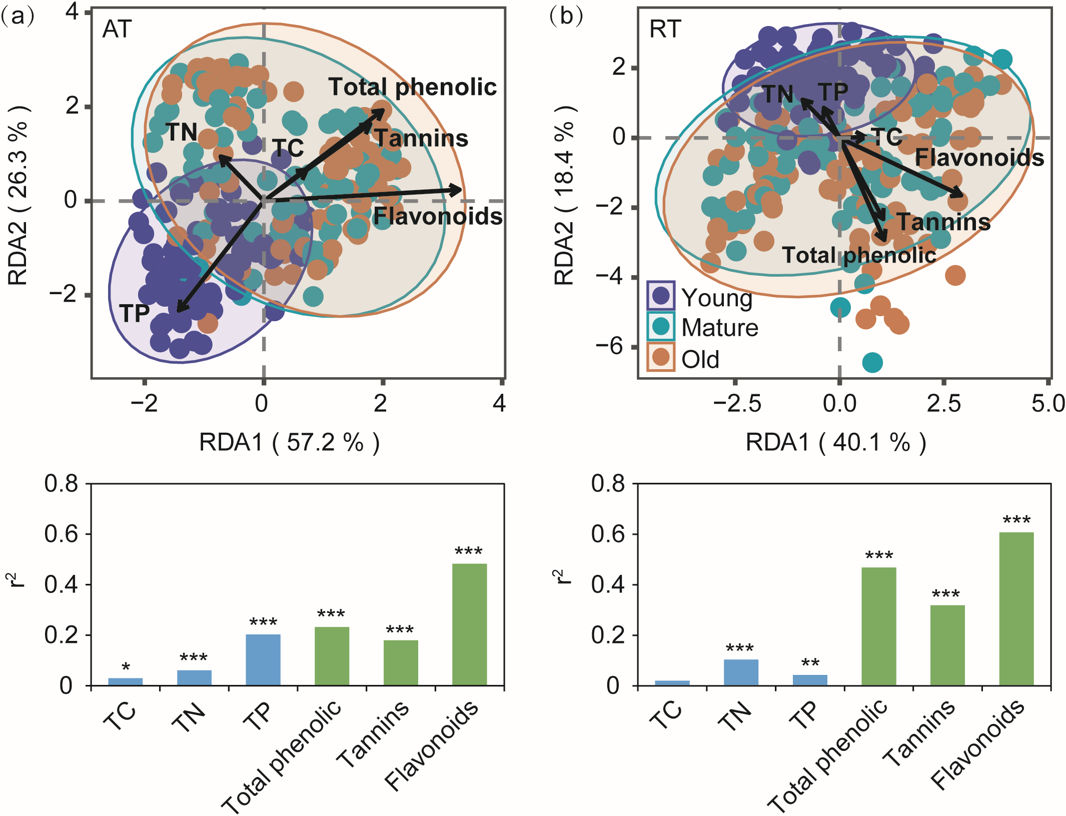
Redundancy analysis (RDA) showing the relative effects of needle economic traits and chemical defense traits on abundant (AT) (**a**) and rare (RT) (**b**) subcommunities. TC, total carbon; TN, total nitrogen; TP, total phosphorus. *P < 0.05; **P < 0.01; ***P < 0.001.

Across all the samples, Pseudomonadota (>55%) and Bacillota (>6%) dominated abundant subcommunities, while Pseudomonadota (>35%), Bacteroidota (>14%), and Actinomycetota (>11%) were the dominant bacterial phyla in rare subcommunities (Fig. S3). For abundant subcommunities, Bacteroidota, Actinomycetota, Planctomycetota, Acidobacteriota, and Deinococcota significantly increased with needle aging, while Pseudomonadota decreased (*P* < 0.001). For rare subcommunities, Abditibacteriota, Acidobacteriota, Actinomycetota, Armatimonadota, Bacteroidota, Bdellovibrionota, Cyanobacteriota, Myxococcota, and Planctomycetota increased with needle aging (*P* < 0.01) (Fig. S3). RDA showed that the needle chemical defense traits, such as flavonoids and total phenolics, explained a relatively higher portion of the variations in both abundant and rare subcommunities compared with the needle economic traits (Fig. 3a and b).

### Assembly patterns of bacterial subcommunities were differentially impacted by needle functional traits

**Fig 4:**
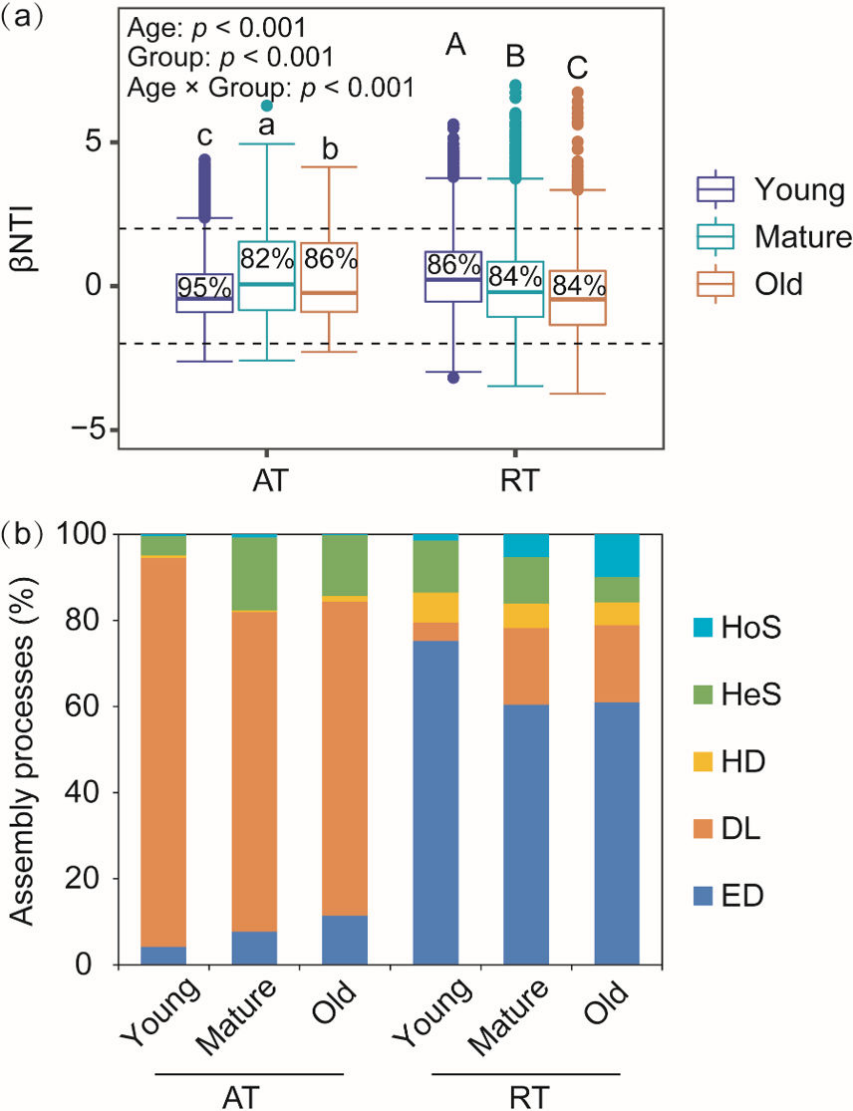
β-nearest taxon index (βNTI) (**a**) and the relative contribution of stochastic (ED, DL, and HD) and deterministic (HeS and HoS) processes (**b**) for abundant (AT) and rare (RT) subcommunities. Percentage represented the proportion of stochastic processes. ED, ecological drift; DL, dispersal limitation; HD, homogenizing dispersal; HeS, heterogeneous selection; HoS, homogeneous selection. Different uppercase and lowercase letters indicated significant differences at *P* < 0.05.

βNTI values showed that the stochastic assembly (>82%) dominated both abundant and rare subcommunities, and deterministic assembly increased in mature and old needles (Fig. 4a). Particularly, DL (>72%) dominated abundant subcommunities and gradually decreased with needle age, while ED and HeS were lower in young needles compared with mature and old needles (Fig. 4b). For rare subcommunities, the assembly was mainly governed by ED (>60%), which performed more in young needles. DL and HoS increased from young needles toward old needles (Fig. 4b). Mantel test results indicated that flavonoids were a valid predictor to the assembly processes of both abundant and rare subcommunities (*P* < 0.01) (Table 1). Moreover, TC:TP and total phenolic affected abundant and rare subcommunities respectively (*P* < 0.05) (Table 1).

**TABLE 1:** Mantel tests of needle functional traits against the β-nearest taxon index (βNTI) of abundant (AT) and rare (RT) subcommunities^*a*^

	AT	RT
TC	0.027	−0.025
TN	−0.080	−0.001
TP	0.020	0.023
TC:TN	−0.023	−0.044
TC:TP	0.048*	0.005
TN:TP	−0.049	0.009
Total phenolic	−0.018	0.045*
Tannins	0.005	0.032
Flavonoids	0.088***	0.057**

^*a*^ Asterisk indicates the significant difference at *P* < 0.05. *P < 0.05; **P < 0.01; ***P < 0.001. TC, total carbon; TN, total nitrogen; TP, total phosphorus.

### Rare subcommunities drove nutrient cycling in phyllosphere habitat

**Fig 5:**
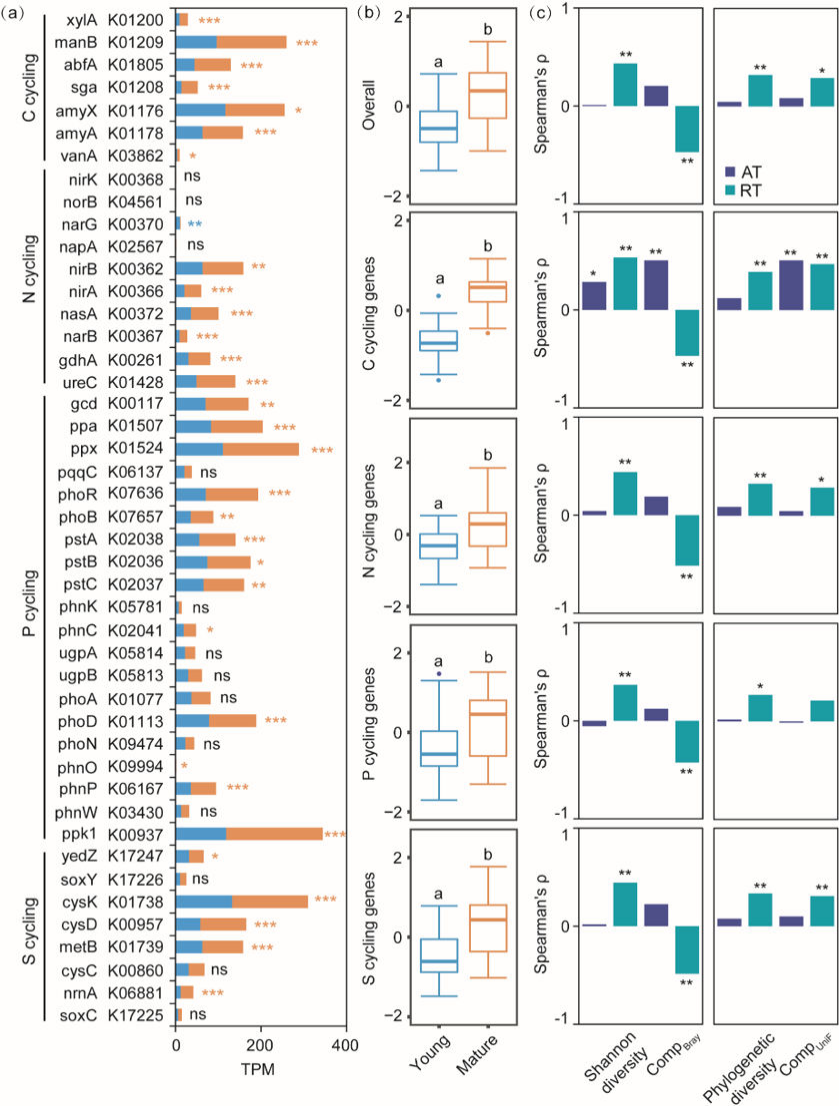
Abundance (TPM) of functional genes involved in C, N, P, and S cycling (**a**). Average abundance (*Z*-score) of overall functional genes and individual functional gene groups (**b**). Different lowercase letters indicated significant differences at *P* <  0.05. Spearman correlation between the average abundance (*Z*-score) of overall functional genes and individual functional gene groups and the properties of abundant (AT) and rare (RT) subcommunities (**c**). Comp_Bray_ and Comp_UniF_ represented the PCoA1 and PCoA2 scores of abundant and rare subcommunities based on the Bray-Curtis distance and Weight-UniFrac distance, respectively. ns, not significant (*P* > 0.05); *P < 0.05; **P < 0.01; ***P < 0.001.

Metagenomic sequencing results indicated that needle age had significant effects on the abundance of functional genes involved in C, N, P, and S cycling (Fig. 5a). The most functional genes related to carbohydrate breakdown (*manB*, *abfA*, *amyX,* and *amyA*), nitrate reduction (*nirB* and *nasA*), organic N metabolism (*gdhA* and *ureC*), inorganic P solubilization (*gcd*, *ppa,* and *ppx*), P transportation (*pstA*, *pstB,* and *pstC*), organic P mineralization (*phoD* and *phnP*), and sulfur-containing amino acid synthesis (*cysK*, *cysD,* and *metB*) were significantly enriched in mature needles, while the abundance of *narG* related to denitrification was higher in young needles than mature needles (Fig. 5a).

Overall, the average abundance of functional genes in mature needles was significantly higher than that in young needles, whether for C, N, P, or S cycling (Fig. 5b). There were significant correlations between the average abundance of different functional gene groups and the diversity and composition of rare subcommunities, whether for taxonomic or phylogenetic level (Fig. 5c). In addition, we showed that the diversity and composition of the abundant subcommunity were significantly related to the average abundance of functional genes involved in C cycling compared with N, P, and S cycling (Fig. 5c).

### Potential functional microorganisms within rare subcommunities

**Fig 6:**
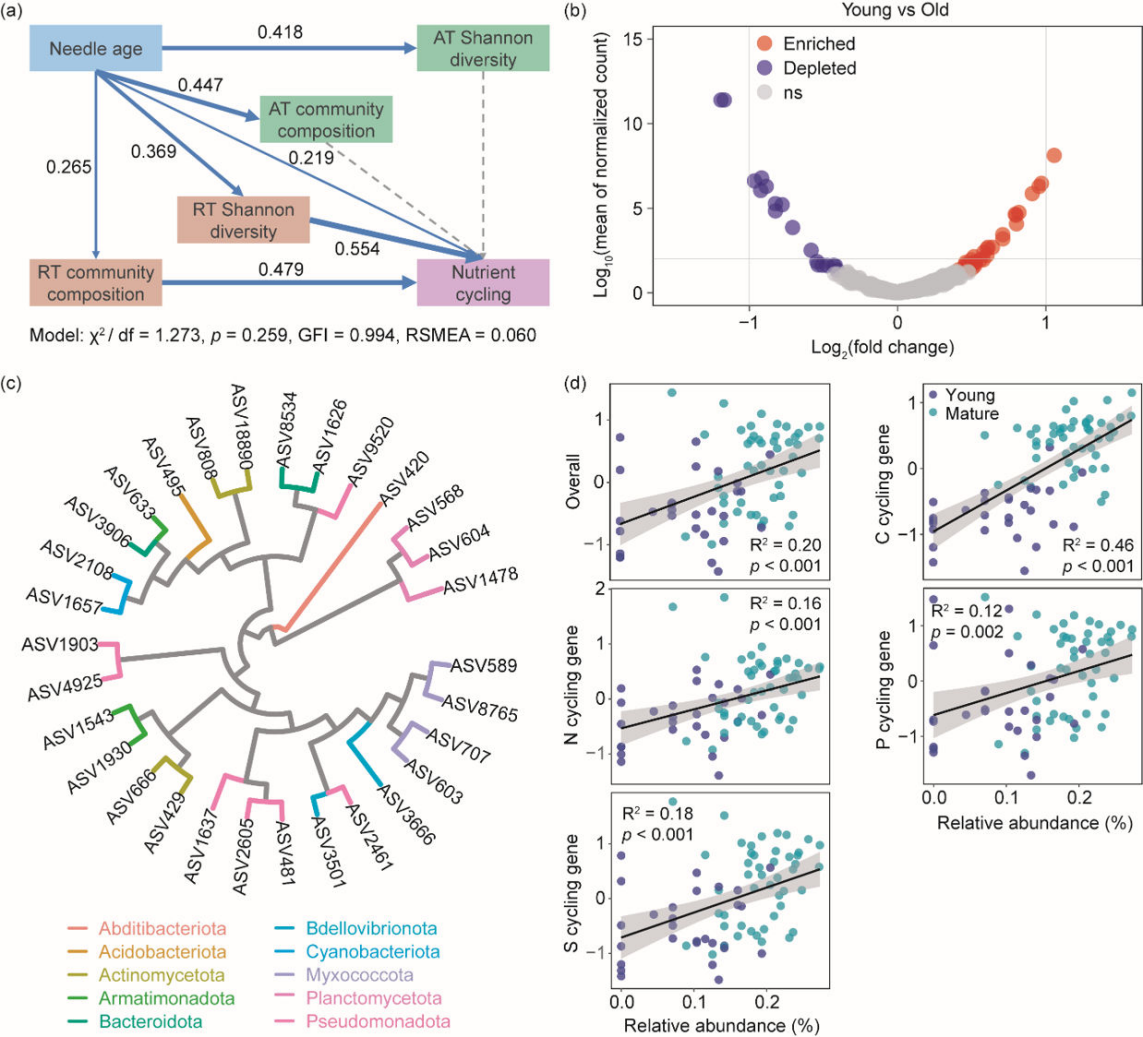
Structural equation model (SEM) was used to explore how abundant (AT) and rare (RT) subcommunities regulated the nutrient cycling in phyllosphere habitat (**a**). AT and RT community composition represented the PCoA1 and PCoA2 scores of abundant and rare subcommunity composition, respectively. Nutrient cycling represented the average abundance (*Z*-score) of overall functional genes involved in C, N, P, and S cycling. Volcano plot was used to identify the biomarker taxa that were enriched in mature needles (**b**). Phylogenetic tree of the biomarker taxa (**c**). Correlations between the relative abundance of biomarker taxa and average abundance (*Z*-scores) of overall functional genes and different functional gene groups (**d**).

SEM provided a good fit to the data (*χ*^2^/df = 1.273, *P* = 0.259, GFI = 0.994, RMSEA = 0.060). It showed that needle age had significant effects on the diversity and composition of both abundant and rare subcommunities. Shifts in rare subcommunities subsequently promoted the average abundance of functional genes involved in C, N, P, and S cycling in phyllosphere habitat, which was also positively influenced by needle aging directly (Fig. 6a). We then identified the biomarker taxa within rare subcommunities, and we found that there were 30 ASVs significantly enriched in mature needles compared with young needles (Fig. 6b).

These dominant ASVs were mainly affiliated within phylum Pseudomonadota (7 ASVs), Actinomycetota (4 ASVs), Myxococcota (4 ASVs), Armatimonadota (3 ASVs), Bacteroidota (3 ASVs), and Planctomycetota (3 ASVs) (Fig. 6c). The dominant taxa belonging to Pseudomonadota were family Beijerinckiaceae and Sphingomonadaceae. The dominant taxa in Bacteroidota were comprised of Chitinophagaceae and Hymenobacteraceae. The families Polyangiaceae and Armatimonadaceae accounted for a high proportion in Myxococcota and Armatimonadota, respectively. There were significant positive correlations between the relative abundance of these biomarker taxa and the abundance of functional genes involved in multiple nutrient cycling (i.e., C, N, P, and S) (Fig. 6d; Fig. S4).

## DISCUSSION

Our knowledge gaps regarding the phyllosphere bacterial communities and plant functional traits have been closed gradually (1, 58). However, the assembly patterns of both abundant and rare bacterial subcommunities, and their relative contributions to nutrient cycling in phyllosphere habitat are still unclear, especially in needles of different ages. The present study revealed that both abundant and rare subcommunities were mainly regulated by stochastic assembly across all needle age cohorts, and deterministic assembly increased with needle aging. Our results also highlighted a key role of rare subcommunities in driving nutrient cycling in phyllosphere habitat of evergreen conifers.

### Stochastic assembly dominated both abundant and rare subcommunities

Determining the processes that regulate microbial communities is crucial to clarify the biodiversity-maintaining mechanisms (59). In contrast to *H*_1_, we found that stochastic assembly was more important for both abundant and rare subcommunities across all needle age cohorts. Particularly, DL dominated the abundant subcommunities, and ED governed the rare subcommunities. These results were consistent with the previous findings reporting that the abundant taxa are more limited by dispersion, whether in agricultural fields, wetlands, or soil-plant continuums (e.g., phyllosphere and rhizosphere) (16, 44, 45). For rare taxa, they are generally recruited through immigration (dispersal events), notably because they have suffered less loss caused by both predation and viral lysis due to their low abundance, and hence hold a potentially unlimited dispersal capacity (60). This phenomenon is particularly common in young leaves with relatively low microbial diversity and abundance (6). In line with this, we found that DL for rare subcommunities was lower in young needles compared with mature and old needles, while ED dominated in young needles.

Our results further indicated that the enhanced deterministic assembly with needle aging for both abundant and rare subcommunities was mainly induced by the increased chemical defense substances, such as flavonoids, in mature and old needles rather than the decreased nutrient content (i.e., P). Plant secondary metabolites have been proven to strongly influence the establishment and assembly of the plant-associated microbiome (61). Flavonoids are important molecules with antimicrobial activities for plant defense against the colonization of microorganisms, especially for pathogenic bacteria (12). Moreover, flavonoids can also play the role of signaling molecules to recruit some specific microbial taxa or act as energy substrates for microbial growth (62). Therefore, these chemical defense compounds can lead to intensified selection effects, especially for the colonization of stress-tolerant taxa, reflected as an apparent increase of Acidobacteriae and Actinomycetota with needle aging (Fig. S2) (63). Overall, these findings provide empirical evidence on the assembly patterns of both phyllosphere abundant and rare bacterial subcommunities and emphasize the importance of needle chemical defense traits in shaping phyllosphere microbial communities of evergreen conifers over time.

### Rare subcommunity drives nutrient cycling in phyllosphere habitat

In line with *H*_2_, both Spearman correlations and SEM showed that the abundance of functional genes related to multiple nutrient cycling (i.e., C, N, P, and S) was positively influenced by the diversity and composition of rare subcommunity. This result highlighted the potential importance of rare subcommunities in regulating multiple nutrient cycling in phyllosphere habitat. This was supported by the recent evidence reporting that the abundant taxa account for most of the biomass and carbon cycling, while the rare taxa are the major drivers of ecosystem multifunctionality (64–66). The positive associations between rare subcommunity diversity and diverse functional genes could arise from the complementarity effects of microbial community due to the niche differences among microbial taxa, which have great potential to promote more functions simultaneously (30). In addition, trait-based microbial strategies, including high yield, resource acquisition, and stress tolerance, have been widely applied in predicting microbial processes (67). Some rare taxa have been proved to be the extremophiles with the greatest potential to take advantage of unique ecological niches and unusual resources, and thereby acting as partners through providing necessary nutrients for other organisms (i.e., complementary metabolic pathways as secretion of vitamins or amino acids) to contribute to geochemical cycling (27, 28, 68, 69). Moreover, stronger deterministic assembly can strengthen the effects of composition changes of rare subcommunities on ecosystem functions when some functional microorganisms were selected and enriched by environmental filtering (70).

In this study, we found that Beijerinckiaceae, Sphingomonadaceae, and Hymenobacteraceae were significantly enriched in mature needles (Fig. S4). Some members of these families are capable of N-fixation and are helpful in increasing N availability in phyllosphere habitat (22, 71). In addition to N fixers, we found that mature needles possessed more abundant Caulobacteraceae, which is well-known for its ability in sulfate metabolism in nutrient-poor environments and is regarded to play a crucial role in ecosystem S cycling and have positive effects for C and N uptake of other taxa (72). Consistent with this, there were significant positive correlations between Caulobacteraceae and multiple S cycling-related functional genes such as *cysK, cysD*, *metB,* and *nrnA* in this study. Moreover, Acidobacteriaceae and Sphingomonadaceae were considered contributors to carbon or energy supply due to their ability in degrading carbohydrate compounds (e.g., xylan) with extracellular enzymes (73). Some members of Myxococcota are well known for their photosynthetic ability and predatory behavior to bacteria. This chimeric lifestyle can make them thrive on oligotrophic foliar surface (74). Besides that, in line with Cheng et al. (75), we also found that Haliangiaceae were closely related to *phoD* and *gcd*, implying that these taxa may participate in promoting P cycling. Collectively, these results highlight that rare subcommunities contribute more to community metabolic potential in phyllosphere habitat of evergreen conifers, and they may directly regulate nutrient cycling dynamics in forest ecosystems. In addition, given the importance of phenolic compounds in shaping phyllosphere microbiomes, together with the differences in functional capability of abundant and rare subcommunities in phenolic biosynthesis, clarifying the potential stress sources and going beyond host plant into future work will further increase our understanding of the mechanisms underlying community assembly and succession of plant-associated microorganisms.

### Conclusions

The results of our field survey provided new insights into the leading role of needle chemical defense traits rather than economic traits in the succession dynamics of both phyllosphere abundant and rare bacterial subcommunities across needle age cohorts of evergreen conifers. We further revealed the importance of variations in diversity and composition of rare subcommunities in driving nutrient cycling in phyllosphere habitat. Overall, identifying the functional taxa within rare subcommunities and taking them into account is necessary when exploring the potential mechanisms underlying the relationships between the host-associated microbial community and forest ecosystem function.

## Data Availability

Needle traits data used in this article are deposited in the Figshare repository (https://doi.org/10.6084/m9.figshare.26156224). Raw sequencing data are archived in the National Microbiology Data Center (https://nmdc.cn) with accession numbers: NMDC10018986.

## AFTER EPUB

*[This article was published on 15 July 2025 with an error in the Acknowledgments. The Acknowledgments were corrected in the current version, posted on 5 August 2025.]*

## SUPPLEMENTAL MATERIAL

Supplemental material spectrum.00518-25-s0001.docx Tables S1 and S2; Fig. S1 to S4.

ASM does not own the copyrights to Supplemental Material that may be linked to, or accessed through, an article. The authors have granted ASM a non-exclusive, world-wide license to publish the Supplemental Material files. Please contact the corresponding author directly for reuse.
