# Supplementary material for: Rare bacterial subcommunity drives nutrient cycling in phyllosphere habitat of evergreen conifers
Source: Microbiol Spectr. 2025 Jul 15;13(8):e00518-25. doi: 10.1128/spectrum.00518-25 (PMC12323584; doi:10.1128/spectrum.00518-25)
Supplement: Supplemental material — Tables S1 and S2; Fig. S1 to S4. [file spectrum.00518-25-s0001.docx]

**Supplementary tables**

**Table S1** Study site description

|  | Location | MAT (℃) | MAP (mm) |
| --- | --- | --- | --- |
| Shengshan (SS) | 126.78°E, 49.48°N | -0.60 | 580.30 |
| Fenglin (FL) | 129.19°E, 48.13°N | 0.94 | 689.62 |
| Liangshui (LS) | 128.90°E, 47.18°N | 1.52 | 693.90 |
| Muling (ML) | 130.16°E, 44.01°N | 3.05 | 653.72 |
| Changbaishan (CBS) | 128.12°E, 42.32°N | 2.78 | 738.55 |

Climate data are collected from WorldClim database for the period of 2010-2019 (<http://www.worldclim.org>). MAT: mean annual temperature; MAP: mean annual precipitation.

**Table S2** Effects of needle age, sampling site, plant species and their interactions on the composition of abundant (AT) and rare (RT) subcommunities tested using PERMANOVA.

|  |  | Bray-Curtis distance | | | Weight-UniFrac distance | | |
| --- | --- | --- | --- | --- | --- | --- | --- |
|  |  | R^2^ | *F* | *p* | R^2^ | *F* | *p* |
| AT | Age | 0.09 | 46.06 | **< 0.001** | 0.11 | 57.70 | **< 0.001** |
|  | Site | 0.19 | 23.83 | **< 0.001** | 0.20 | 25.76 | **< 0.001** |
|  | Species | 0.13 | 31.82 | **< 0.001** | 0.12 | 29.83 | **< 0.001** |
|  | Age × Site | 0.01 | 1.78 | **0.005** | 0.02 | 2.32 | **< 0.001** |
|  | Age × Species | 0.02 | 4.88 | **< 0.001** | 0.02 | 5.89 | **< 0.001** |
|  | Site × Species | 0.08 | 5.29 | **< 0.001** | 0.07 | 4.90 | **< 0.001** |
|  | Age × Site × Species | 0.02 | 1.69 | **0.001** | 0.03 | 2.00 | **< 0.001** |
| RT | Age | 0.02 | 7.94 | **< 0.001** | 0.05 | 18.08 | **< 0.001** |
|  | Site | 0.10 | 8.27 | **< 0.001** | 0.11 | 10.10 | **< 0.001** |
|  | Species | 0.07 | 11.02 | **< 0.001** | 0.08 | 14.43 | **< 0.001** |
|  | Age × Site | 0.02 | 1.98 | **< 0.001** | 0.02 | 2.08 | **< 0.001** |
|  | Age × Species | 0.02 | 3.00 | **< 0.001** | 0.02 | 3.15 | **< 0.001** |
|  | Site × Species | 0.08 | 3.91 | **< 0.001** | 0.07 | 3.79 | **< 0.001** |
|  | Age × Site × Species | 0.03 | 1.46 | **< 0.001** | 0.03 | 1.42 | **0.003** |

**Supplementary figures**


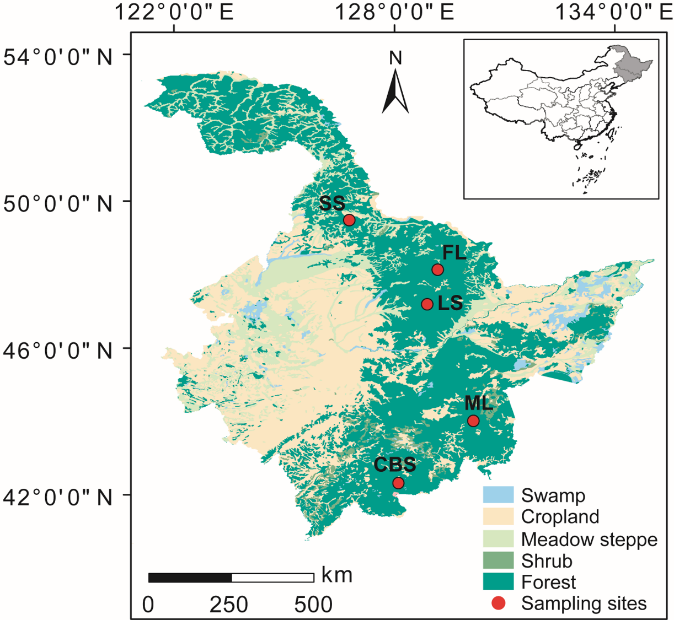


**Fig. S1** Location of sampling sites. Vegetation dataset was collected from the National Cryosphere Desert Data Center.


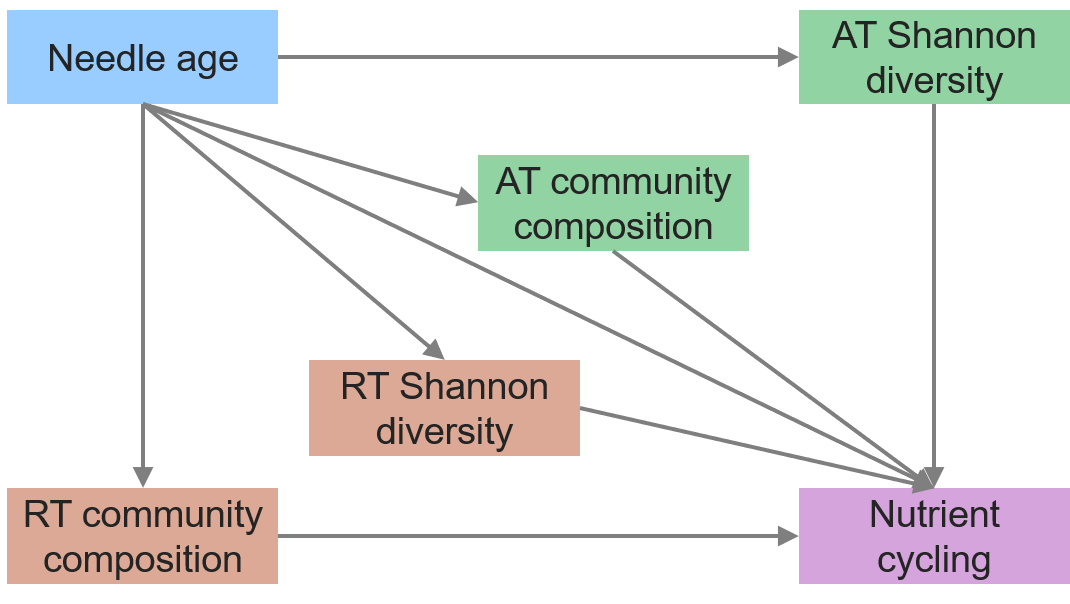


**Fig. S2** A priori conceptual structural equation model (SEM) depicting pathways by which needle age, diversity and composition of abundant and rare subcommunities may influence nutrient cycling in phyllosphere habitat. The single-headed arrows indicate a hypothesized causal effect of one variable on another.


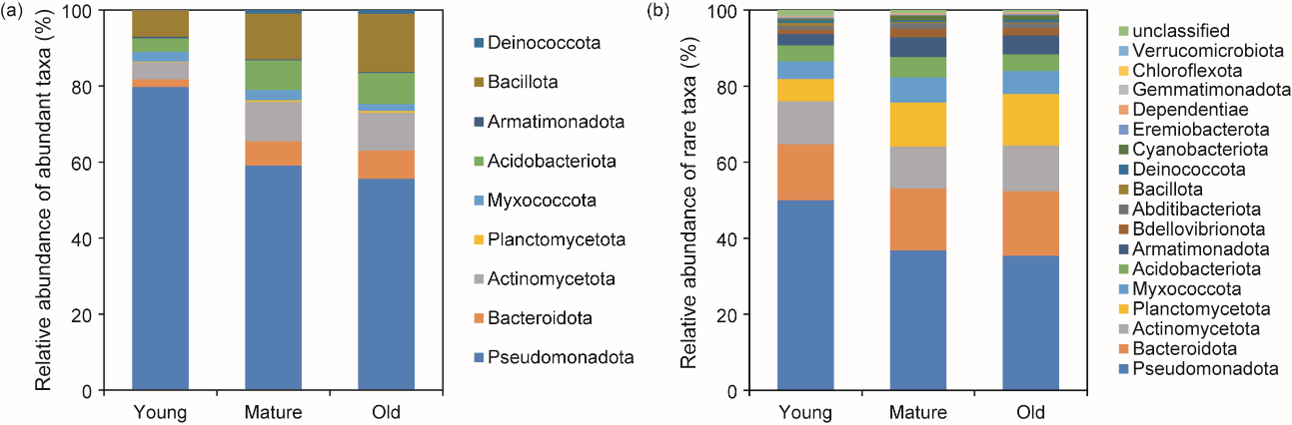


**Fig. S3** Relative abundance (%) of abundant (a) and rare (b) taxa at the phylum level.


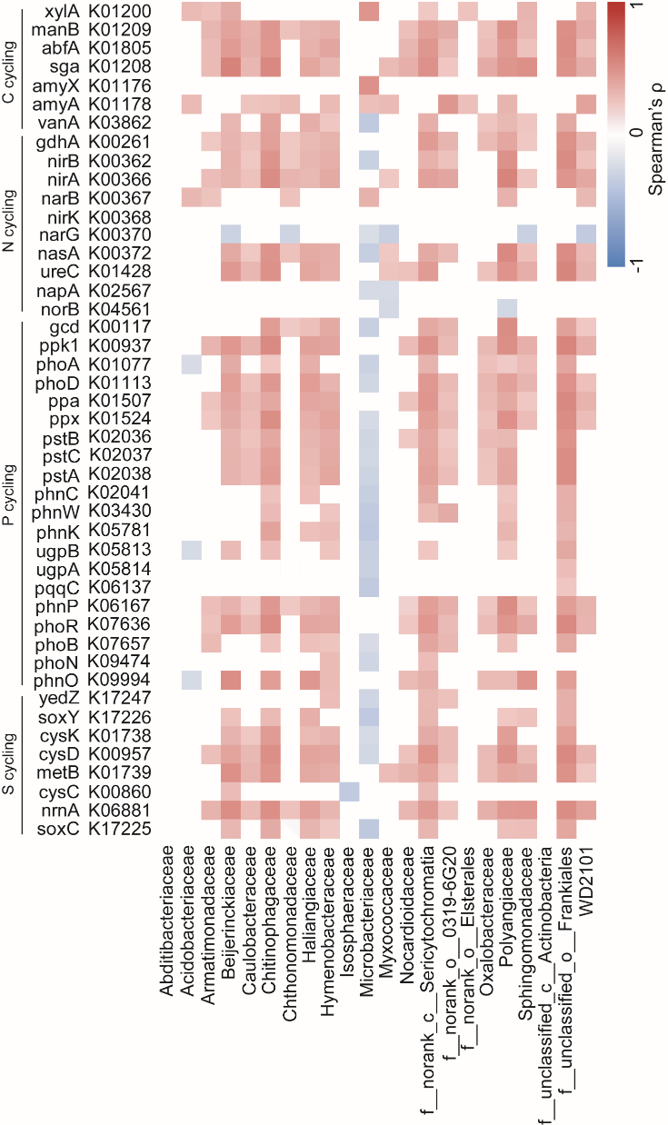


**Fig. S4** Spearman correlations between the abundance of functional genes and the relative abundance of rare taxa those were enriched in mature needles at the family level. Significant correlations (*p* < 0.05) were showed.
